# Supplementary figures and images for: Strain phylogroup and environmental constraints shape Escherichia coli dynamics and diversity over a 20-year human gut time series
Source: ISME J. 2024 Dec 12;19(1):wrae245. doi: 10.1093/ismejo/wrae245 (PMC11728103; doi:10.1093/ismejo/wrae245)

Tree scale: 0.01

### Dates in days

0  
694  
1389  
2084  
2779  
3474  
4169  
4864  
5559  
6254  
6949

### Phylogroups

A  
B1  
B2  
D  
E  
F  
G  
H

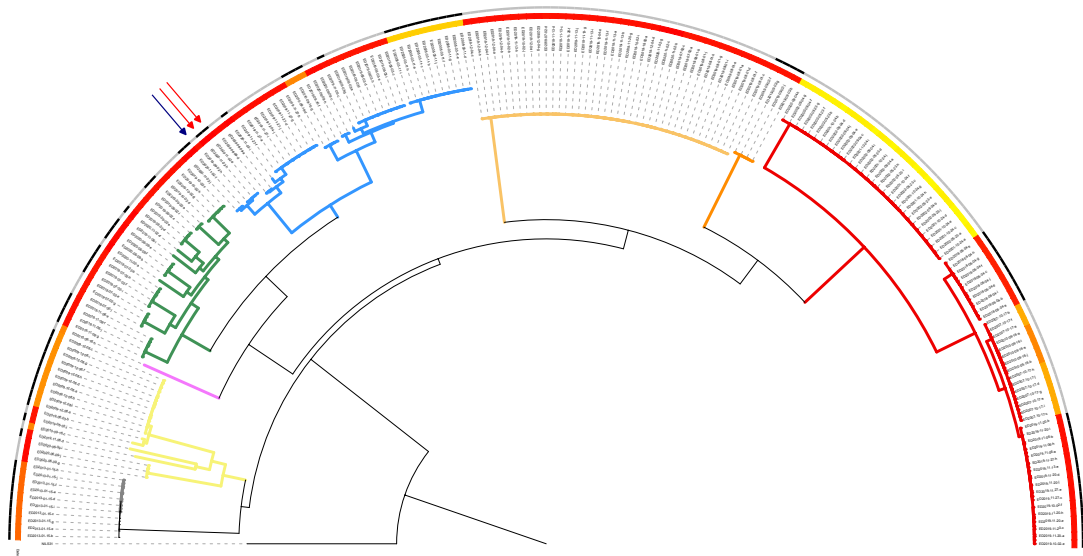

Supplement: FigS1_wrae245 [file figs1_wrae245.pdf]

SNPs in core genome

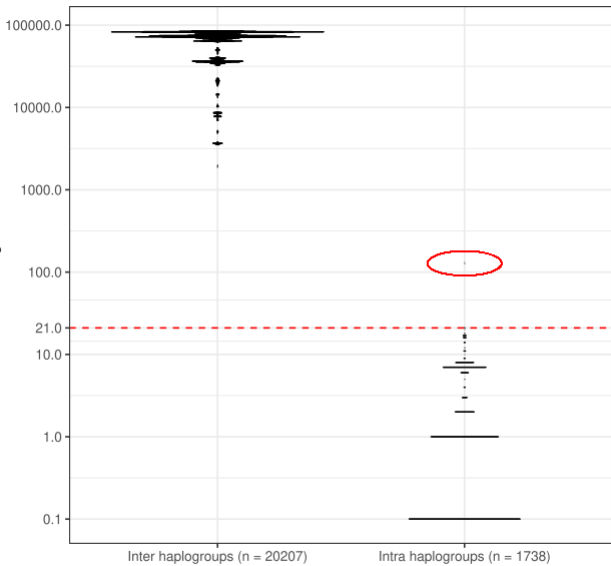

Supplement: FigS2_wrae245 [file figs2_wrae245.pdf]

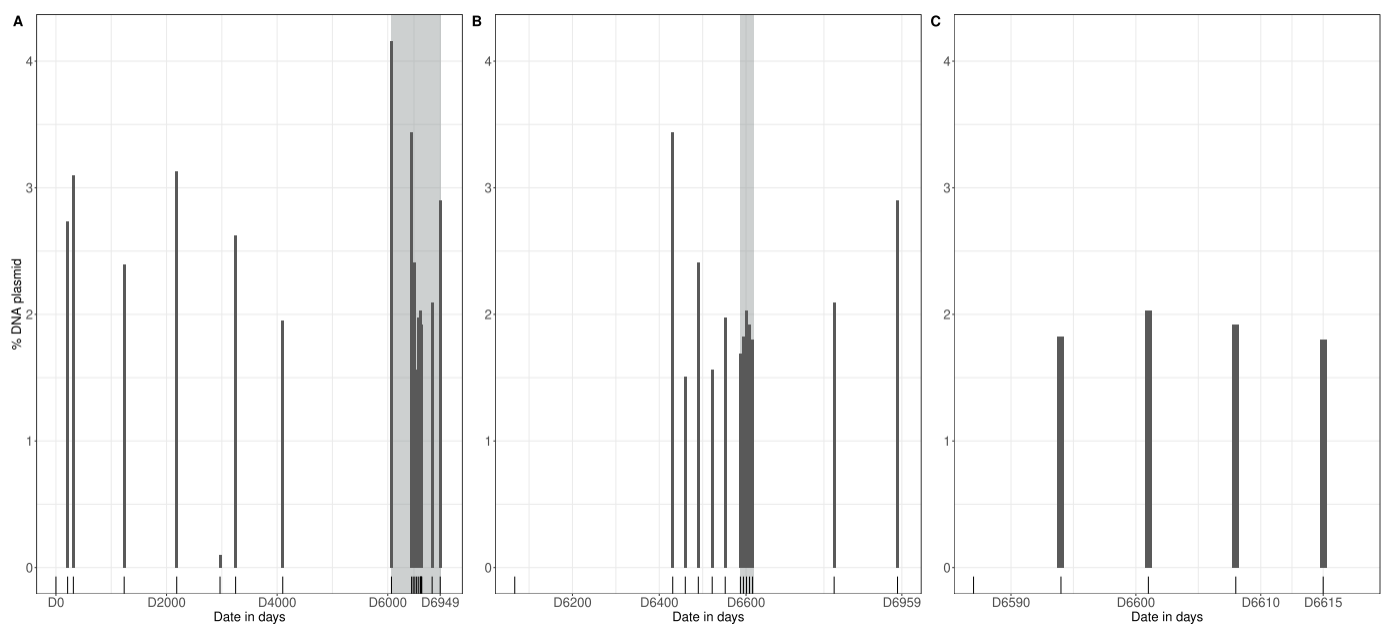

Supplement: FigS3_wrae245 [file figs3_wrae245.pdf]

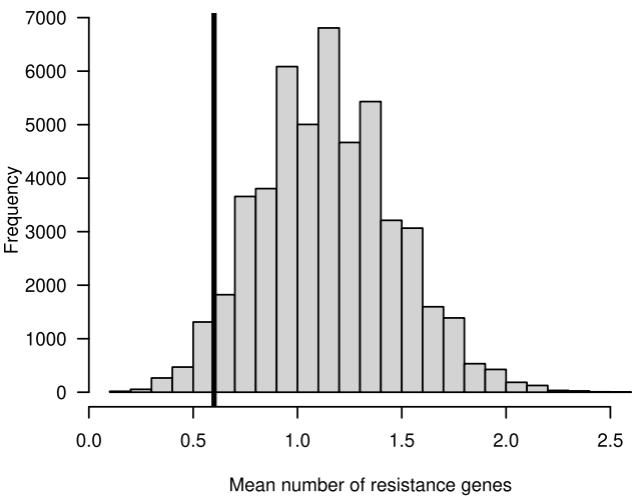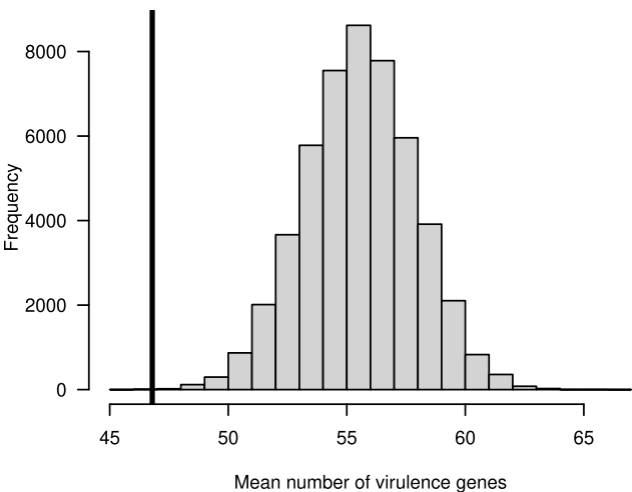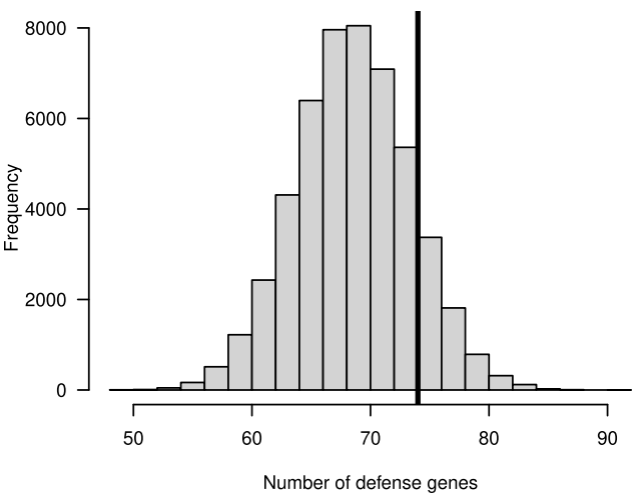

Supplement: FigS4_wrae245 [file figs4_wrae245.pdf]

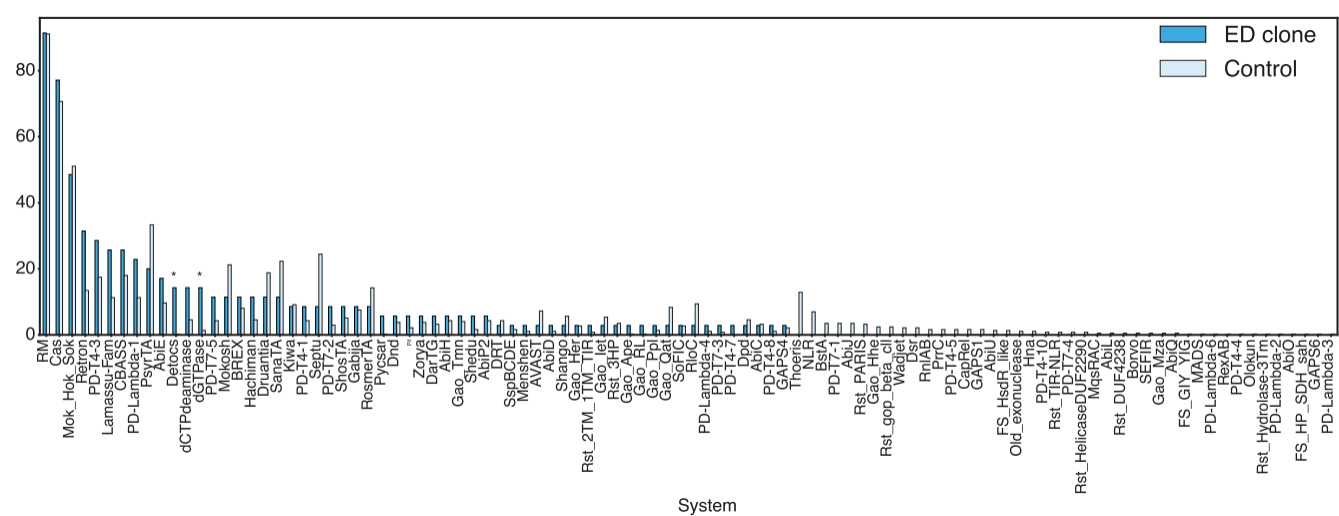

Supplement: FigS5_wrae245 [file figs5_wrae245.pdf]

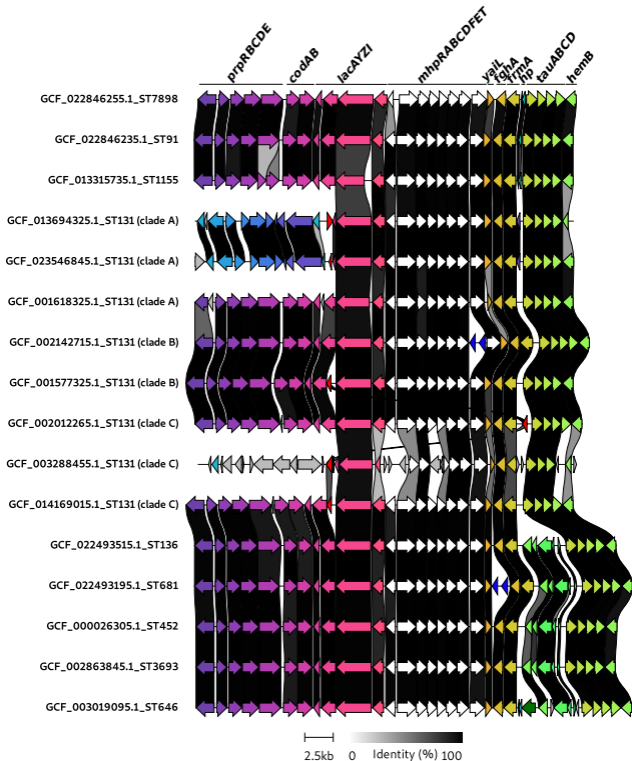

Supplement: FigS6_wrae245 [file figs6_wrae245.pdf]
