# Supplementary material for: Strain phylogroup and environmental constraints shape Escherichia coli dynamics and diversity over a 20-year human gut time series
Source: ISME J. 2024 Dec 12;19(1):wrae245. doi: 10.1093/ismejo/wrae245 (PMC11728103; doi:10.1093/ismejo/wrae245)
Supplement: FigS7_wrae245 [file figs7_wrae245.pdf]

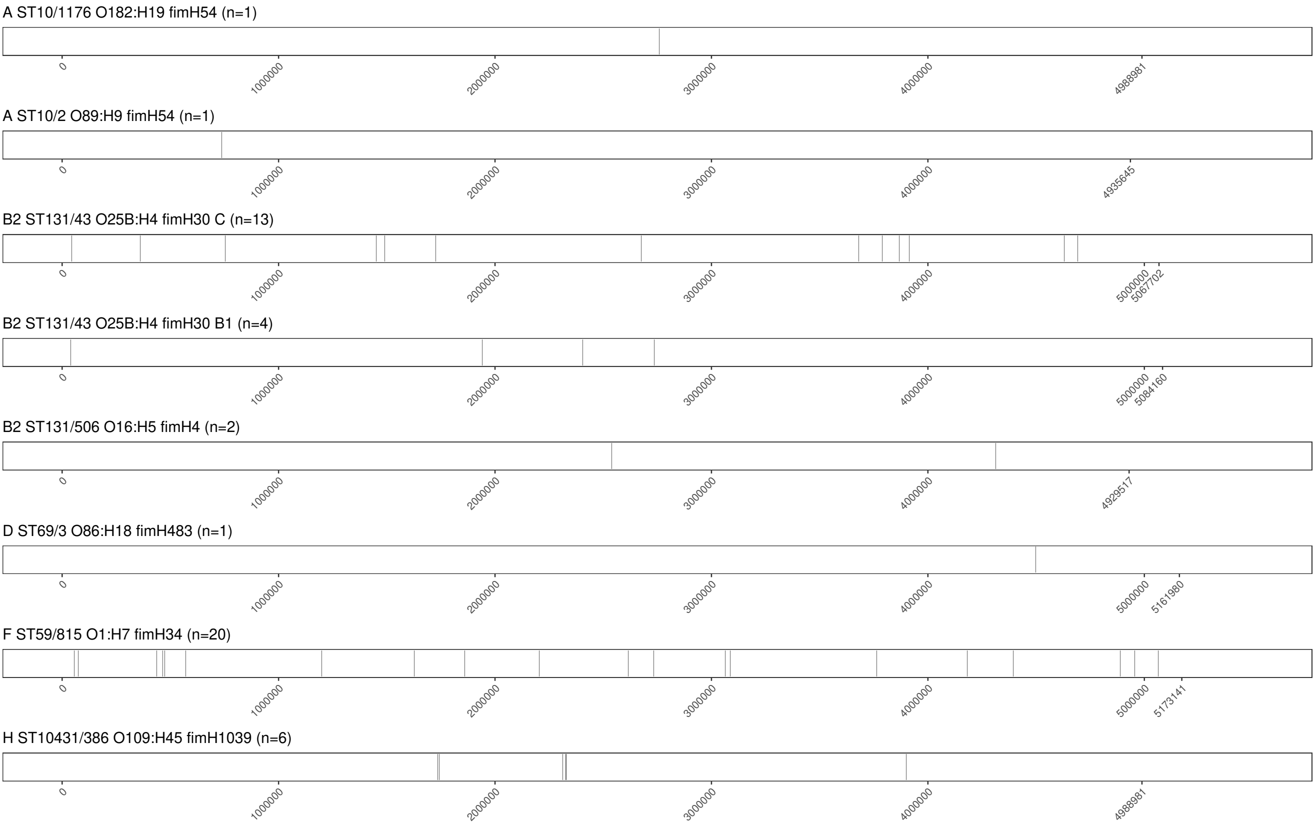
A ST10/1176 O182:H19 fimH54 (n=1)
0
1000000
2000000
3000000
4000000
4988981
A ST10/2 O89:H9 fimH54 (n=1)
0
1000000
2000000
3000000
4000000
4935645
B2 ST131/43 O25B:H4 fimH30 C (n=13)
0
1000000
2000000
3000000
4000000
5000000
5067702
B2 ST131/43 O25B:H4 fimH30 B1 (n=4)
0
1000000
2000000
3000000
4000000
5000000
5084160
B2 ST131/506 O16:H5 fimH4 (n=2)
0
1000000
2000000
3000000
4000000
4929517
D ST69/3 O86:H18 fimH483 (n=1)
0
1000000
2000000
3000000
4000000
5000000
5161980
F ST59/815 O1:H7 fimH34 (n=20)
0
1000000
2000000
3000000
4000000
5000000
5173141
H ST10431/386 O109:H45 fimH1039 (n=6)
0
1000000
2000000
3000000
4000000
4988981
