# Supplementary material for: Strain phylogroup and environmental constraints shape Escherichia coli dynamics and diversity over a 20-year human gut time series
Source: ISME J. 2024 Dec 12;19(1):wrae245. doi: 10.1093/ismejo/wrae245 (PMC11728103; doi:10.1093/ismejo/wrae245)
Supplement: m_s_supp_isme_j-d-24-00345_wrae245 [file m_s_supp_isme_j-d-24-00345_wrae245.docx]

**Revised version ISMEJ-D-24-01008**

**The following presents supplementary online material (SOM) of the article “Strain phylogroup and environmental constraints shape *Escherichia coli* dynamics and diversity over a twenty-year human gut time series” by:**

Bénédicte Condamine^1^, Thibaut Morel-Journel^1,2^, Florian Tesson^1, 3^, Guilhem Royer^1,4,5^, Mélanie Magnan^6^, Aude Bernheim^3^, Erick Denamur^1,7*^, François Blanquart^8*^, Olivier Clermont^1*^

^1^ Université Paris Cité, INSERM, IAME, 75018 Paris, France

^2^ Université Sorbonne Paris Nord, INSERM, IAME, 93000 Bobigny, France

^3^ Institut Pasteur, Université Paris Cité, INSERM, Molecular Diversity of Microbes Lab, 75015 Paris, France.

^4^ Unité de Bactériologie, Département de Prévention, Diagnostic et Traitement des Infections, AP-HP, Hôpital Henri Mondor, Créteil, France.

^5^ EA 7380 Dynamyc, EnvA, UPEC, University of Paris-Est, Créteil, France

^6^ Université Paris Cité, INSERM, CNRS, Institut Cochin, UMR 1016, 75014, Paris, France.

^7^ AP-HP, Laboratoire de Génétique Moléculaire, Hôpital Bichat, 75018 Paris, France

^8^ Center for Interdisciplinary Research in Biology, CNRS, Collège de France, PSL Research University, 75005 Paris, France

* ED, FB and OC shared last authorship on this work

Corresponding author: Erick Denamur, [erick.denamur@inserm.fr](mailto:erick.denamur@inserm.fr)

**Material/Subject and methods**

*Description of the ED subject*

ED stands for the initials of the subject. ED is a caucasian man, married with children, living and working in Paris. He was 44 year-old at the beginning of the study, weighing around 65 kg for a height of 1.75 m. Importantly, he did not take any antibiotics nor had been hospitalized during the sampling period. He is omnivorous with few (twice a week) meat and fish consumption, regular (daily) vegetable and fruit consumption and enrichment in dairy products and cereal (every breakfast) intake. He drinks a cup of wine every day. In a simplified dietary questionnaire designed to characterize the diet quality of the French population [[1]](https://www.zotero.org/google-docs/?Z9jaUl), ED scored the highest for cereal consumption (1/day) and dairy products (1/day). He traveled on average once a year outside France (Europe, USA, China, Australia) for short periods (less than two weeks). He never had a pet.

In sum, ED can be globally considered as representative of the healthy French urban adult population, with some diet specificities.

*Stool plating and isolate sampling*

Fecal samples were stored at 4°C after emission and transmitted to the laboratory. An aliquot of the fresh feces (one inoculation loop) was spread on Drigalski agar plates pure or sterile water diluted at 10^-2^ to obtain at least 10 isolated colonies. The plates were incubated at 37°C overnight (O/N), and the feces were discarded. Ten colonies (called “isolate” in the main text) with a yellow appearance were isolated from each plate at each time point. These colonies were then grown on liquid lysogeny broth (LB) O/N and stored at −80°C in glycerol. We choose to study 10 colonies because this level of depth is often used in the literature and it represents a good trade-off between the detection of diversity and the amount of work and money spent.

*Interpretation of the multiple occurrences of ST399Cplx clones and the* yjcB-ssb *defense island in the ED subject*

The *yjcB-ssb* defense island detection within 1,777 RefSeq complete genomes, was based on the detection of both flanking genes of more than 300 bp of the island (*pdeC* and *uvrA* genes).

We detail calculations to infer the minimal number of *E. coli* cells contending to colonize a focal host per unit time, based on the repeated observation over ∆t = 427 days of clones of *E. coli* of ST399 Cplx carrying the anti-phage defense island *yjcB-ssb*. We denote each genotype by two letters: S or T, corresponding to clones of the focal subtype (ST399 Cplx) or not; and D or E, corresponding to clones with the defense island or not.

In the table below, we compare the number of observed genotypes $N_{i,j}$ (where the subscript *i, j* denotes the genotype), with the frequencies of these genotypes $X_{i,j}$ in three reference datasets: our control commensal collection, the RefSeq database (high-quality genomes), and 70,301 genomes from Enterobase. These reference datasets are assumed to roughly represent the frequency of these genotypes in the bacterial pool to which the subject is exposed.

| Genotype | Observed  $N_{i,j}$ | Commensal  $X_{i,j}$ | RefSeq  $X_{i,j}$ | Enterobase  $X_{i,j}$ | Commensal  ratio  $N_{i,j}/X_{i,j}$ | RefSeq ratio$N_{i,j}/X_{i,j}$ | Enterobase ratio $N_{i,j}/X_{i,j}$ |
| --- | --- | --- | --- | --- | --- | --- | --- |
| S,D | 5 | 0 | 1/1777 | 57/70301 | max 598* | 8885 | 6167 |
| S,E | 1 | 4/359 | 14/1777 | 56/70301 | 90 | 127 | 1255 |
| T,D | 0 | 0 | 1/1777 | 55/70301 | 0 | 0 | 0 |
| T,E | 12 | 355/359 | 1761/1777 | 70133/70301 | 12 | 12 | 12 |

* the frequency in commensal was 0, and was conservatively replaced with an upper bound on the frequency calculated with the rule of three in order to get non-infinite result.

Assuming the rate of contact of the subject ED with *E. coli* cells is $\beta$, a fraction $\varepsilon_{i,j}$ of these events is successful, a fraction $\omega$ is observed with our experimental design, the rates of observed colonization by each genotype are:

$\beta\varepsilon_{i,j}\omega X_{i,j}$

The total number of colonization events observed over ∆t $N_{tot}$, , thus follows a Poisson distribution with rate $\beta\omega\Delta t\sum_{i,j} \varepsilon_{i,j}{{X_{i,j}}}$. The frequencies of the different clones among successful colonization events follows a multinomial distribution with parameters $\varepsilon_{i,j}X_{i,j}/\sum_{i,j} \varepsilon_{i,j}X_{i,j}$ and $N_{tot}$ . The maximum likelihood estimators of unknown parameters $\beta$ , $\omega$, $\varepsilon_{i,j}$satisfy:

$\beta\omega\Delta t\varepsilon_{i,j}{{X_{i,j}}}= N_{i,j} \forall i,j$

We obtain a lower bound for the rate of incoming *E. coli* clones $\beta$ by setting $\omega=1$ (all clones are detected) and $\varepsilon_{i,j}=1$ for over-represented genotypes (these genotypes always successfully colonize). This leads to the following lower bound for the parameter $\beta$ :

$\beta\geq\frac{N_{i,j}}{X_{i,j}} \Delta$t^-1^

The best lower bound for the rates of incoming clones is given by the genotype with the highest $N_{i,j}/X_{i,j}$ ratio (Table), in our case S,D. This lower bound is equal to 1.4 d^-1^, 20 d^-1^, and 14 d^-1^ in each of the three reference datasets respectively.

To conclude, the occurrence of clones of the ST399 Cplx carrying the defense island can only be achieved if ED is exposed to at least 1 to 20 bacterial cells from this pool per day. This lower bound could be improved if we had more complete knowledge of the detection rate of clones with our sampling design (parameter $\omega$).

mhp *gene cluster detection and analysis*

We searched for the presence of the *mph* gene cluster in the 1777 RefSeq genomes using a custom database for abricate (https://github.com/tseemann/abricate) with identity and coverage thresholds of 90%. We considered the presence of the cluster only in genomes carrying all genes (i.e. 8 genes : *mhpRABCDEFT*). Then, sequences were extracted using samtools v1.18 [[2]](https://www.zotero.org/google-docs/?jFJ1dg) and aligned with mafft v7.490 [[3]](https://www.zotero.org/google-docs/?OjmbzA). We computed a maximum likelihood phylogenetic tree from the alignment using iq-tree v1.6.12 [[4]](https://www.zotero.org/google-docs/?LVOnJV) with the option “MF” to select for the best-fit model (i.e. TIM3+F+R2).

We then compared the evolutionary history of *mhp* with that of the B2 phylogroup strains. In brief, we constructed a pangenome using Ppanggolin [[5]](https://www.zotero.org/google-docs/?g0OCiL) including all RefSeq B2 strains (n=282/1777), ED1a (NC_011745.1) and a genome belonging to phylogroup G (GCF_001900735.1) as outgroup. Then, we computed a maximum likelihood phylogenetic tree from core gene alignment of these 284 genomes with iq-tree with the option “MF” to select for the best-fit model (*i.e.* GTR+F+R4). We determined multi-locus sequence types using MLST (<https://github.com/tseemann/mlst>) and the scheme of the University of Warwick [[6]](https://www.zotero.org/google-docs/?vC7UDd). Finally, the tree was annotated using Itol [[7]](https://www.zotero.org/google-docs/?2c4BH8).

To analyze the genetic environment of the *mhp* gene cluster among B2 strains, we extracted nucleotide sequences ranging from *prpR* to *hemB* genes among all B2 genomes. In the absence of *prpR*, we extracted sequences up to 16,771 bp upstream of *mhpR* to obtain an equivalent distance. Then, we clustered sequences using CD-HIT [[8]](https://www.zotero.org/google-docs/?cmSYab) with identity threshold of 99% and coverage of 95% and kept one represent for each cluster. From the 16 sequences obtained, we construct a physical map using clinker [[9]](https://www.zotero.org/google-docs/?AhBQ08) using standard parameters. We annotated the map with the sequence type of the strains and, in the case of ST131, assigned the strains to t clades A (O16:H5/*fimH41*, B (O25B:H4/*fimH161*, O25B:H4/*fimH22*) or C (O25B:H4/*fimH41*).

*Within-clone diversity*

We assessed the within-clone diversity as the divergence created by the mutations accumulated by each clone during its residence in ED. Conversely to genetic diversity across clones, we were here specifically interested in the differences observed between isolates of the same clone. Therefore, we considered here the clones sampled more than once (two to seven times, for a total of 16 samples considered).

Among those clones, F ST59/815 O1:H7 *fimH*34 presented two distinct haplotypes, both already present in the first sample and differing from each other by six mutations. Both haplotypes were also found in later isolates (the first one 92, 134, 141 and 155 days later; the second one 30 and 489 days later), so that they coexisted in the gut. We performed a clustering analysis on the profiles of all isolates from this clone in order to assign them to one of the two groups, and we assessed the number of differences within each group, compared with the difference between them. The between-cluster variance represented 67.9% of the total, and 1.6 and 1.1 mutations respectively separated two isolates from the same cluster on average, while 7.4 mutations separated two clones belonging to different clusters. Moreover, the six mutations identified in the first sample never appeared in the first group, but always in the second. For the following analyses, we therefore considered these two haplotypes separately.

First, we estimated *ν* the average observed mutation rate of these clones in ED, i.e. excluding highly deleterious mutations that would not be observed. To do so, we computed the average number of mutations separating two isolates of the same sample *i* (noted $\Pi_{i,j}$) and of two separate samples *i* and *j* (noted $\Pi_{i,j}$ ). The number of observed mutations accumulated per site over *T* days separating the two samples ($T=j-i$) could be estimated as $\nu T = \Pi_{ij}- \Pi_{ii}$ [[10]](https://www.zotero.org/google-docs/?UZhiI8). We computed $\nu T$ for the 12 samples that were not the first for a given clone (58 isolates), and a linear regression over time was performed to estimate the observed mutation rate per site per day.

Second, we simulated the expected divergence between isolates of the same clone over time under neutrality, using a Wright-Fisher model. Starting from two identical genotypes at time$t=0 (\Pi_{00}=0)$, the average number of mutations separating two isolates is expected to increase towards the mutation-drift equilibrium, depending only on the mutation rate per site per generation (noted *μ*) and the effective population size (noted $N_{e}$) [[10]](https://www.zotero.org/google-docs/?hWtBL3). To assess those transient dynamics and compare them to our data, we performed these simulations over 1065 days (the longest time between two samples of the same clone in ED) for various population sizes $N_{e}$, ranging from 100 to 10000.

# **References**

[1. Chaltiel D, Adjibade M, Deschamps V, Touvier M, Hercberg S, Julia C, et al. Programme National Nutrition Santé - guidelines score 2 (PNNS-GS2): development and validation of a diet quality score reflecting the 2017 French dietary guidelines. *Br J Nutr* 2019; 122: 331–342.](https://www.zotero.org/google-docs/?7Z6H7m)

[2. Danecek P, Bonfield JK, Liddle J, Marshall J, Ohan V, Pollard MO, et al. Twelve years of SAMtools and BCFtools. *GigaScience* 2021; 10: giab008.](https://www.zotero.org/google-docs/?7Z6H7m)

[3. Katoh K, Standley DM. MAFFT multiple sequence alignment software version 7: improvements in performance and usability. *Mol Biol Evol* 2013; 30: 772–780.](https://www.zotero.org/google-docs/?7Z6H7m)

[4. Nguyen L-T, Schmidt HA, von Haeseler A, Minh BQ. IQ-TREE: a fast and effective stochastic algorithm for estimating maximum-likelihood phylogenies. *Mol Biol Evol* 2015; 32: 268–274.](https://www.zotero.org/google-docs/?7Z6H7m)

[5. Gautreau G, Bazin A, Gachet M, Planel R, Burlot L, Dubois M, et al. PPanGGOLiN: Depicting microbial diversity via a partitioned pangenome graph. *PLoS Comput Biol* 2020; 16: e1007732.](https://www.zotero.org/google-docs/?7Z6H7m)

[6. Wirth T, Falush D, Lan R, Colles F, Mensa P, Wieler LH, et al. Sex and virulence in Escherichia coli: an evolutionary perspective. *Mol Microbiol* 2006; 60: 1136–1151.](https://www.zotero.org/google-docs/?7Z6H7m)

[7. Letunic I, Bork P. Interactive Tree of Life (iTOL) v6: recent updates to the phylogenetic tree display and annotation tool. *Nucleic Acids Res* 2024; gkae268.](https://www.zotero.org/google-docs/?7Z6H7m)

[8. Fu L, Niu B, Zhu Z, Wu S, Li W. CD-HIT: accelerated for clustering the next-generation sequencing data. *Bioinforma Oxf Engl* 2012; 28: 3150–3152.](https://www.zotero.org/google-docs/?7Z6H7m)

[9. Gilchrist CLM, Chooi Y-H. clinker & clustermap.js: automatic generation of gene cluster comparison figures. *Bioinforma Oxf Engl* 2021; 37: 2473–2475.](https://www.zotero.org/google-docs/?7Z6H7m)

[10. Fu YX. Estimating mutation rate and generation time from longitudinal samples of DNA sequences. *Mol Biol Evol* 2001; 18: 620–626.](https://www.zotero.org/google-docs/?7Z6H7m)

**Supplementary online material (SOM)**

**Supplementary text Materials/subjects and methods**

**Supplementary Tables**

Table S1. Main characteristics of the 210 *E. coli* ED isolates

Table S2. ST distribution of the 210 *E. coli* ED isolates

Table S3. SNPs matrix of 210 *E. coli* ED isolates

Table S4. Main characteristics of the 35 *E. coli* ED clones

### Table S5. List of genes significantly associated to the 35 ED clones

Table S6. Distribution of *mhp* gene cluster among 1,777 complete *E. coli* genomes from RefSeq

Table S7. List of mutations identified in nine *E. coli* ED clones corresponding to 128 isolates

**Supplementary Figures**

##

## **Fig. S1. Maximum likelihood phylogenetic tree reconstructed from the core genome single nucleotide polymorphisms (SNPs) (n=3 217 191) of the 210 *E. coli* ED isolates.**

The three isolates of the ST399 O25B (ED2020-11-02-e and ED2020-11-02-h) and ST399 O25A (ED2019-09-02-h) are indicated by red and blue arrows, respectively.

## **Fig. S2. Distribution of the pairwise core genome SNP distances according to whether pairs of isolates are inter- or intra-haplogroup***.*

## For clarity, the SNP scale is logarithmic with 0.1 for 0. The red dotted line corresponds to the cutoff chosen for the definition of clones. The dots circled in red correspond to the phylogroup A_ ST399/661_O25:H12_fimHND haplogroup isolates differing by 125-130 SNPs.

## **Fig. S3. Mean of the cumulative number of replicons per sample of the 210 *E. coli* ED isolates over time.** Panels A, B and C panels correspond to the yearly, monthly and weekly samples, respectively, as in Fig. 1.

**Fig. S4. Distribution of resistance, virulence genes and anti-phage defense systems between ED clones and commensal strains.** Mean number of resistant and virulence genes, and total number of defense genes in the 35 ED clones (vertical lines), together with the null distribution when sampling 35 strains at random among the 359 commensals of the control collection (histograms)**.** The ED clones count slightly less resistant genes and less virulence genes, but a similar number of defense systems. The sampling of the control collection was done 50,000 times.

##

**Fig. S5**. **Percentage of strains encoding each defense system between the ED clones and the control commensal collection.** Only two systems are significantly more abundant in the ED clones (chi2 test corrected by Bonferroni, significance threshold *p*  < 0.05): dGTPase and Detocs.

**Fig. S6. Physical map of the *mhp* cluster and its genetic environment among phylogroup B2 representative genomes.** Representative genomes from phylogroup B2 were selected from the RefSeq dataset for each *mhp* environment based on nucleotide clustering (identity=99%, coverage=95%). The ED1a ST452 genome (GCF_000026305.1) was also included. White arrows represent the *mhp* gene cluster. Genes that are homologous between different genomes are of the same colour. The links between the maps represent the identity of the proteins. Insertion sequences are highlighted in red (IS*1*), blue (IS*3*) and green (IS*110*). The combination of serotypes and *fimH* allele was used to assign genomes belonging to ST131 to the clades A (O16:H5*/fimH41*, B (O25B:H4/*fimH161,* O25B:H4/*fimH22*) or C (O25B:H4/*fimH41*).

**Fig. S7. Schematic representation of the SNPs identified in the eight clone isolates sequenced by Illumina and Nanopore technologies along their chromosomes.** The origin (0) corresponds to the *dnaA* gene. The scale represents 1 million bp. The total number of SNPs is indicated with the name of the clone. The SNPs are indicated by vertical bars.
